# Predicting epilepsy after new onset refractory status epilepticus due to autoimmune encephalitis: The DAME score

**DOI:** 10.1002/epi.70081

**Published:** 2025-12-30

**Authors:** Simona Lattanzi, Sara Matricardi, Alberto Vogrig, Giada Pauletto, Margherita Nosadini, Stefano Sartori, Federico Massa, Luana Benedetti, Stefano Meletti, Francesca Bisulli, Elena Freri, Francesca Felicia Operto, Silvia Bozzetti, Sara Mariotto, Simone Beretta, Eleonora Rosati, Elisabetta Cesaroni, Carla Marini, Tiziana Granata, Flavio Villani, Immune Epilepsies Study Group of the Italian League Against Epilepsy, Ruggero Bernabei, Stefania Maria Bova, Silva Cappanera, Luigi Caputi, Edward Cesnik, Francesco Deleo, Lidia Di Vito, Giuseppe D’Orsi, Giovanni Falcicchio, Elisa Fallica, Edoardo Ferlazzo, Lucia Fusco, Matteo Gastaldi, Giada Giovannini, Angela La Neve, Claudio Liguori, Pietro Mattioli, Lorenzo Muccioli, Carlotta Mutti, Angelo Pascarella, Elena Pasini, Daniela Passarelli, Francesca Ragona, Romana Rizzi, Elena Tartara, Valentina Tontini, Alberto Verrotti, Federica Zibordi, Lucia Zinno, Antonio Zito

**Affiliations:** ^1^ Neurological Clinic, Department of Experimental and Clinical Medicine Polytechnic University of Marche Ancona Italy; ^2^ Department of Pediatrics University of Chieti Chieti Italy; ^3^ Department of Medicine University of Udine Udine Italy; ^4^ Clinical Neurology, Department of Head‐Neck and Neuroscience Azienda Sanitaria Universitaria Friuli Centrale Udine Italy; ^5^ Pediatric Neurology and Neurophysiology Unit, Department of Women's and Children's Health, University Hospital of Padua, Neuroimmunology Group Pediatric Research Institute “Città Della Speranza” Padua Italy; ^6^ Department of Neuroscience, Rehabilitation, Ophthalmology, Genetics, Maternal and Child Health University of Genoa Genoa Italy; ^7^ IRCCS Ospedale Policlinico San Martino Genoa Italy; ^8^ Department of Biomedical Metabolic Sciences and Neurosciences University of Modena and Reggio Emilia Reggio Emilia Italy; ^9^ Neurophysiology Unit and Epilepsy Center, Neuroscience Department Modena AOU Modena Italy; ^10^ IRCCS Istituto Delle Scienze Neurologiche di Bologna, full member of European Reference Network EpiCARE Bologna Italy; ^11^ Department of Biomedical and Neuromotor Sciences University of Bologna Bologna Italy; ^12^ Department of Pediatric Neuroscience Fondazione IRCCS Istituto Neurologico Carlo Besta, full member of European Reference Network EpiCARE Milan Italy; ^13^ Department of Health Sciences, School of Medicine University Magna Graecia of Catanzaro Catanzaro Italy; ^14^ Department of Neurology/Stroke Unit San Maurizio Hospital, Südtiroler Sanitätsbetrieb Bolzano Italy; ^15^ Paracelsus Medical University Salzburg Austria; ^16^ Neurology Unit, Department of Neurosciences, Biomedicine, and Movement Sciences University of Verona Verona Italy; ^17^ Department of Neurology Fondazione IRCCS San Gerardo Dei Tintori Monza Italy; ^18^ Department of Medicine and Surgery University of Milano‐Bicocca Milan Italy; ^19^ Emergency Neurology, Neuroscience Department Careggi University Hospital Florence Italy; ^20^ Child Neurology and Psychiatry Unit Children's Hospital “G. Salesi,” Ospedali Riuniti Ancona Ancona Italy; ^21^ Division of Clinical Neurophysiology and Epilepsy Center IRCCS Ospedale Policlinico San Martino Genoa Italy

**Keywords:** autoimmune encephalitis, etiology, new onset refractory status epilepticus, status epilepticus

## Abstract

**Objective:**

This study aimed to identify risk factors and develop a predictive scoring system for autoimmune‐associated epilepsy in subjects with autoimmune encephalitis presenting with new onset refractory status epilepticus (NORSE).

**Methods:**

This retrospective, multicenter, cohort study included subjects who presented with NORSE at the onset of autoimmune encephalitis and had at least 24 months of follow‐up after immunotherapy. The outcome was the development of autoimmune‐associated epilepsy, defined as persistent seizures despite adequate immunotherapy and absence of active inflammation. Factors independently associated with the outcome were identified through a backward stepwise selection. Adjusted regression coefficients of each independent predictor were transformed to produce a points‐based risk‐scoring system.

**Results:**

Seventy participants were included (median age = 24.2 years, 38.6% male). During a median follow‐up of 53 months, 54.3% of subjects developed autoimmune‐associated epilepsy. Status epilepticus duration ≥ 10 days (odds ratio [OR] = 31.14, 95% confidence interval [CI] = 2.12–456.87, *p* = .012), positivity for antibodies against surface antigens (OR = .12, 95% CI = .02–.85, *p* = .034), bitemporal magnetic resonance imaging (MRI) abnormalities suggestive of autoimmune encephalitis during acute stage (OR = 49.80, 95% CI = 2.95–841.77, *p* = .007), and interictal epileptiform discharges during electroencephalographic (EEG) follow‐up (OR = 71.32, 95% CI = 6.48–785.32, *p* < .001) were independently associated with the study outcome. The duration–antibodies–MRI–EEG (DAME) score was developed as an integer‐based scoring system predictive of autoimmune‐associated epilepsy. With an optimal cutoff of ≥3 points, it yielded a sensitivity of 86.8%, a specificity of 87.5%, and an overall accuracy of 87.1%.

**Significance:**

The DAME score could serve as a user‐friendly score to predict the risk of autoimmune‐associated epilepsy in patients with NORSE due to autoimmune encephalitis.

**Key points**

- The DAME score predicts the risk of epilepsy in patients with autoimmune encephalitis‐associated NORSE.
- The score consists of four variables: duration of status epilepticus, antibody status, MRI abnormalities, and interictal EEG findings.
- The score yields a sensitivity of 86.8%, a specificity of 87.5%, and an accuracy of 87.1%.
- The score may inform prognosis prediction, customization of follow‐up, and improvement of long‐term management.

## 1. INTRODUCTION

Most autoimmune encephalitis present with seizures, and some cases evolve to long‐term epilepsy. The Autoimmunity and Inflammation Task Force of the International League Against Epilepsy (ILAE) defined “autoimmune‐associated epilepsy” as persistent seizures despite adequate immunotherapy and in the absence of clear evidence of active inflammation.^1^ The effect of inflammation in these subjects is irreversible and results in an enduring predisposition to spontaneous seizures.

New onset refractory status epilepticus (NORSE) is a rare, challenging clinical presentation characterized by the occurrence of refractory status epilepticus (SE) with no readily identifiable cause in otherwise healthy individuals.^2^ NORSE is a heterogeneous condition associated with distinct prognosis and a variety of underlying etiologies. Functional disability and subsequent chronic epilepsy are common after NORSE, but some people have good outcomes.^3^ Interestingly, autoimmune encephalitis represents one of the most commonly identified causes of NORSE,^4^ and refractory SE has been suggested to act as a risk factor for epileptogenesis and drug‐resistant epilepsy in subjects with autoimmune encephalitis.^5^, ^6^

In a previous multicentric cohort study, we described the epileptic phenotypes and long‐term outcomes of 263 participants with new onset seizures in the context of autoimmune encephalitis.^7^ To expand the results further, in the current analysis, we aimed to identify the risk factors and develop a predictive scoring system for autoimmune‐associated epilepsy in subjects with NORSE due to autoimmune encephalitis.

## 2. MATERIALS AND METHODS

### 2.1. Study setup and data collection

This was a subgroup analysis with extended follow‐up of a previously published retrospective, multicenter cohort study performed at 34 Italian epilepsy centers and including patients who experienced epileptic seizures at the onset or during the acute phase of autoimmune encephalitis.^7^ Only subjects presenting with NORSE at the onset of autoimmune encephalitis and with a minimum follow‐up of 24 months after immunotherapy were considered in the current analysis.

The diagnosis of autoimmune encephalitis was defined according to consensus criteria in adult^8^ and pediatric patients,^9^ including the following: (1) definite antibody‐positive autoimmune encephalitis and (2) probable antibody‐negative autoimmune encephalitis. Patients with prior history of epilepsy or with comorbidities known to confer a significant risk of developing seizures (e.g., alcohol or drug abuse, previous brain lesions) were excluded.

Medical charts at admission and during follow‐up visits were reviewed to collect demographic and clinical data, including age, gender, duration, and type (i.e., with prominent motor symptoms or nonconvulsive) of SE. Paraclinical findings, including cerebrospinal fluid (CSF) analysis, electroencephalography (EEG), and brain neuroimaging, were also reviewed. All paired serum and CSF samples were tested for an antibody panel using commercial kits. In cases where results were nondiagnostic, additional investigations were carried out through fixed and live cell‐based assays, tissue‐based assays, and immunoblot, following standardized protocols and expert guidelines, to detect the presence of antineuronal antibodies. Details of collected information were already described elsewhere.^7^

The outcome was the development of autoimmune‐associated epilepsy, defined by the ILAE as persistent seizures despite adequate immunotherapy and in the absence of clear evidence of active inflammation.^1^ The diagnosis of autoimmune‐associated epilepsy and the time definition of the acute phase or relapses of autoimmune encephalitis were determined by consensus according to a combination of clinical evidence of active encephalitis and paraclinical findings.^1^, ^7^

### 2.2. Statistical analysis

Values are presented as median (interquartile range [IQR]) for continuous variables and as number (percent) of subjects for categorical variables. Comparisons were made through Mann–Whitney test or chi‐squared test. Age, gender, and characteristics associated with outcome in the univariate analysis with *p* ≤ .05 were entered into the multiple logistic regression model. A backward stepwise selection with variable entry set at *p* = .05, and removal at *p* = .10 was adopted to identify factors independently associated with the outcome. Any continuous variable included in the multiple logistic regression model was first converted to a categorical variable based on the optimal cut point estimated in the cohort through receiver operating characteristic (ROC) curve and Youden index. Adjusted regression coefficients of each independent predictor were transformed to produce a points‐based risk‐scoring system; the weighted score of each factor was obtained by dividing each adjusted regression coefficient by the smallest one and rounding it to the nearest integer. Any negative score was indicative of a negative (protective) association with the outcome.

Discrimination of the model (i.e., the ability of a model to differentiate between individuals who developed or did not develop epilepsy) was measured using the ROC curve. Calibration (i.e., the agreement between the predicted and observed risk of epilepsy) was assessed with the calibration plot. Perfect calibration is implied by a 45° diagonal line; relevant deviations above or below reflect underprediction or overprediction.

### 2.3. Standard protocol approvals, registrations, and patient consent

This study has been approved by the Regional Ethics Committee of Liguria, Italy (ID 12278). All participants or their legal representatives provided written informed consent according to the Declaration of Helsinki.

## 3. RESULTS

There were 79 subjects who presented with NORSE of 263 cases initially identified.^7^ After the exclusion of subjects who died (*n* = 7) or were lost to follow‐up (*n* = 2) within the first 24 months, 70 participants were included.

The median age at NORSE onset was 24.2 (IQR = 8–50) years, and 27 (38.6%) were males; 30 patients (42.9%) were of pediatric (<18 years old) age (median age = 6.3 years, IQR = 4–9.1) and 40 (57.1%) of adult age (median age = 42.5 years, IQR = 29–65). SE with prominent motor symptoms and nonconvulsive SE were diagnosed in 53 (75.7%) and 17 (24.3%) cases, respectively. The median duration of SE was 19 (IQR = 10–37) days.

Antineuronal antibodies were identified in 30 (42.9%) patients, of whom 27 had antibodies targeting neuronal cell‐surface antigens, and three had antibodies against intracellular antigens. Details of the antibodies identified are summarized in Table S1. No antineuronal antibodies were identified in the remaining 40 (57.1%) cases, which met the inclusion criteria for probable antibody‐negative autoimmune encephalitis. Brain magnetic resonance imaging (MRI) during the acute phase revealed abnormalities suggestive of autoimmune encephalitis (T2/fluid‐attenuated inversion recovery [FLAIR] hyperintensities) restricted to one (*n* = 10, 14.3%) or both (*n* = 16, 22.9%) temporal lobes, in extratemporal areas or multifocally in gray matter, white matter, or both (*n* = 22, 31.4%). Patients received immunotherapy after a median delay from onset of 7 (IQR = 5–15) days. All patients received first‐line treatment (e.g., intravenous methylprednisolone, intravenous immunoglobulins, plasma exchange), and second‐line treatment (e.g., rituximab, cyclophosphamide, anakinra, tocilizumab) was administered in 17 (24.3%) cases. Second‐line immunotherapies were prescribed in 11 of 27 (40.7%) subjects with autoimmune encephalitis and antibodies against surface antigens, five of 40 (12.5%) subjects with seronegative autoimmune encephalitis, and one of three (33.3%) subjects with autoimmune encephalitis and antibodies against intracellular antigens.

During a median follow‐up of 53 (IQR = 40–90) months, 38 of 70 (54.3%) subjects developed autoimmune‐associated epilepsy. The duration of follow‐up was 46.5 (IQR = 39.5–78.5) and 60 (IQR = 40–126) months in people with and without epilepsy, respectively (*p* = .354). Follow‐up EEG identified interictal epileptiform discharges (IEDs) in 38 (54.3%) subjects. Characteristics of study participants and univariate associations with the development of autoimmune‐associated epilepsy are shown in Table 1. The proportions of subjects who developed autoimmune‐associated epilepsy according to the identified antibodies are provided in Table S1. At the last follow‐up visit, all patients who were diagnosed with epilepsy were receiving antiseizure medication (ASM) treatment, as were nine of 32 (28.1%) of the patients who were not.

**TABLE 1:** Characteristics of study participants and associations with the development of autoimmune‐associated epilepsy.

Characteristic	All participants, *n* = 70	Autoimmune‐associated epilepsy		OR (95% CI); *p*
		No, *n* = 32	Yes, *n* = 38	
Age, years	24.2 (8–50)	15.9 (4.5–42.5)	32.5 (10–53)	1.01 (.99–1.03); *p* = .194
Female, *n* (%)	43 (61.4)	18 (56.3)	25 (65.8)	1.50 (.57–3.94); *p* = .415
Status epilepticus semiology, *n* (%)				
With prominent motor symptoms	53 (75.7)	21 (65.6)	32 (84.2)	2.79 (.90–8.71); *p* = .077
Nonconvulsive	17 (24.3)	11 (34.4)	6 (15.8)	.36 (.11–1.12); *p* = .077
Duration of status epilepticus, days	19 (10–37)	12 (5.5–22.5)	27 (12–48)	1.04 (1.01–1.07); *p* = .009
Serum and/or CSF antineuronal antibodies, *n* (%)				
Antibodies to surface antigens	27 (38.6)	19 (59.4)	8 (21.1)	.18 (.06–.52); *p* = .002
Antibodies to intracellular antigens	3 (4.3)	2 (6.3)	1 (2.6)	.41 (.04–4.69); *p* = .470
Antibody negative	40 (57.1)	11 (34.4)	29 (76.3)	6.15 (2.16–17.49); *p* = .001
CSF findings, *n* (%)				
Lymphocytic pleocytosis, >5 cells/dL	34 (48.6)	16 (50.0)	18 (47.4)	.90 (.35–2.31); *p* = .826
Elevated protein, >50 mg/dL	24 (34.3)	10 (31.3)	14 (36.8)	1.28 (.47–3.48); *p* = .624
Intrathecal synthesis of oligoclonal bands	18 (25.7)	8 (25.0)	10 (26.3)	1.07 (.36–3.15); *p* = .900
Brain MRI findings, *n* (%)^a^				
Temporal unilateral	10 (14.3)	5 (15.6)	5 (13.2)	.82 (.21–3.12); *p* = .769
Temporal bilateral	16 (22.9)	1 (3.1)	15 (39.5)	20.22 (2.49–164.25); *p* = .005
Extratemporal or multifocal in gray and/or white matter	22 (31.4)	9 (28.1)	13 (34.2)	1.33 (.48–3.69); *p* = .585
Time to immunotherapy, days	7 (5–15)	5.5 (3–11)	9 (6–21)	1.03 (.99–1.07); *p* = .205
Second‐line treatment, *n* (%)	17 (24.3)	11 (34.4)	6 (15.8)	.36 (.11–1.12); *p* = .077
Follow‐up				
Interictal epileptiform discharges, *n* (%)	38 (54.3)	7 (21.9)	31 (81.6)	15.82 (4.90–51.10); *p* < .001
Brain MRI atrophy, *n* (%)	18 (25.7)	5 (15.6)	13 (34.2)	2.81 (.87–9.01); *p* = .083

*Note*: Data are median (interquartile range) for continuous variables and *n* (%) for categorical variables.

Abbreviations: CI, confidence interval; CSF, cerebrospinal fluid; MRI, magnetic resonance imaging; OR, odds ratio.

^a^ T2/fluid‐attenuated inversion recovery hyperintensity compatible with inflammation.

SE duration was converted to a categorical variable based on the estimated optimal cut point of 10 days. In the multivariate regression analysis, independent risk factors for the development of epilepsy were the following: SE duration ≥ 10 days (odds ratio [OR] = 31.14, 95% confidence interval [CI] = 2.12–456.87, *p* = .012), bitemporal MRI abnormalities suggestive of autoimmune encephalitis during the acute stage (OR = 49.80, 95% CI = 2.95–841.77, *p* = .007), and IEDs during follow‐up (OR = 71.32, 95% CI = 6.48–785.32, *p* < .001); contrariwise, the positivity for antibodies directed against surface antigens was independently associated with a lower likelihood of autoimmune‐associated epilepsy (OR = .12, 95% CI = .02–.85, *p* = .034).

Based on these findings, we created the duration–antibodies–MRI–EEG (DAME) score, an integer‐based scoring system predictive of autoimmune‐associated epilepsy. The DAME score integrated SE duration ≥ 10 days, the presence of antibodies against surface antigens, T2/FLAIR hyperintensities of both temporal lobes during the acute stage of autoimmune encephalitis, and IEDs at follow‐up EEG (Table 2).

**TABLE 2:** The DAME score.

Variable	DAME score, points
Duration of status epilepticus ≥ 10 days	
No	0
Yes	+2
Antibodies against surface antigens	
No	0
Yes	−1
MRI bitemporal abnormalities^a^	
No	0
Yes	+2
EEG interictal epileptiform discharges	
No	0
Yes	+2

*Note*: To calculate an individual's DAME score, the points associated with each predictor can be added to obtain the total risk score. The score can range from a minimum of −1 to a maximum of 6 points. Abbreviations: DAME, duration–antibodies–MRI–EEG; EEG, electroencephalography; MRI, magnetic resonance imaging.

^a^ T2/FLAIR hyperintensity compatible with inflammation.

The associations of the different parameters of the score with the development of autoimmune‐associated epilepsy according to the antibody status are summarized in Table S2. Duration of SE ≥ 10 days, bitemporal MRI abnormalities, and EEG IEDs were associated with the risk of autoimmune‐associated epilepsy in subjects either with or without identified antibodies.

**FIGURE 1:**
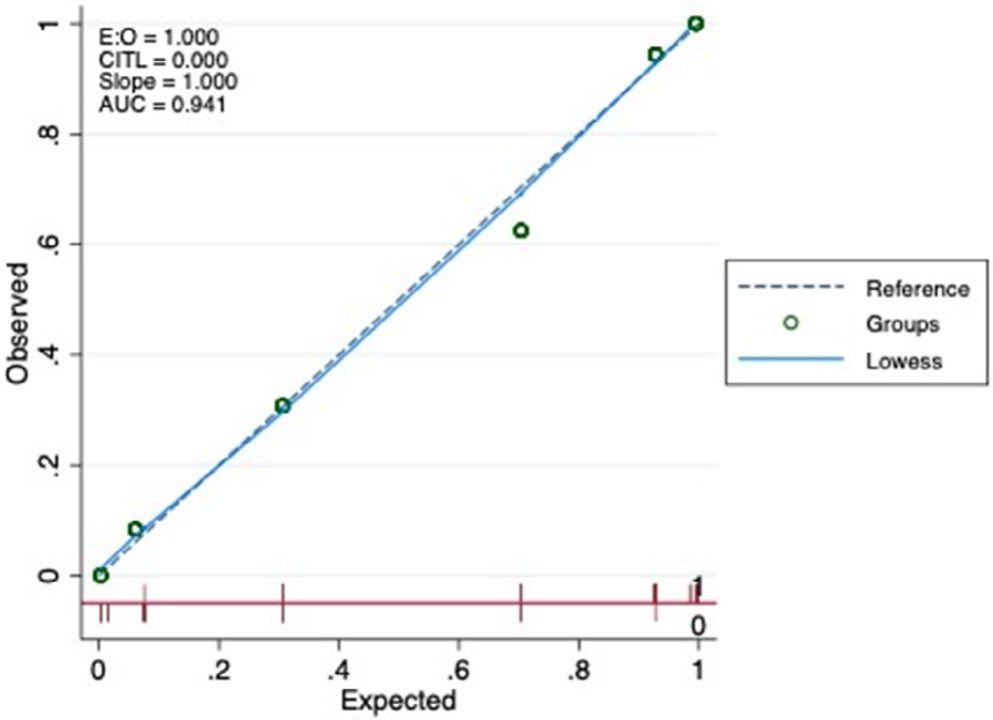
Calibration plot for predicting autoimmune‐associated epilepsy. Observed probability of autoimmune‐associated epilepsy is plotted against predicted probability of autoimmune‐associated epilepsy. Diagonal line indicates perfect calibration. AUC, area under the curve; E:O, Ratio (Events/Observations); CITL, Calibration in the Large.

The area under the curve (AUC) of the score was .941 (95% CI = .891–.990), and no statistically significant differences were found in the discrimination ability of the score across the adult and pediatric populations (AUC_adults_ = .906, 95% CI = .824–.989 and AUC_children_ = .969, 95% CI = .905–1.000; *p* for DeLong test = .239). Calibration plot indicated good fit of predicted and observed data (Figure 1).

**FIGURE 2:**
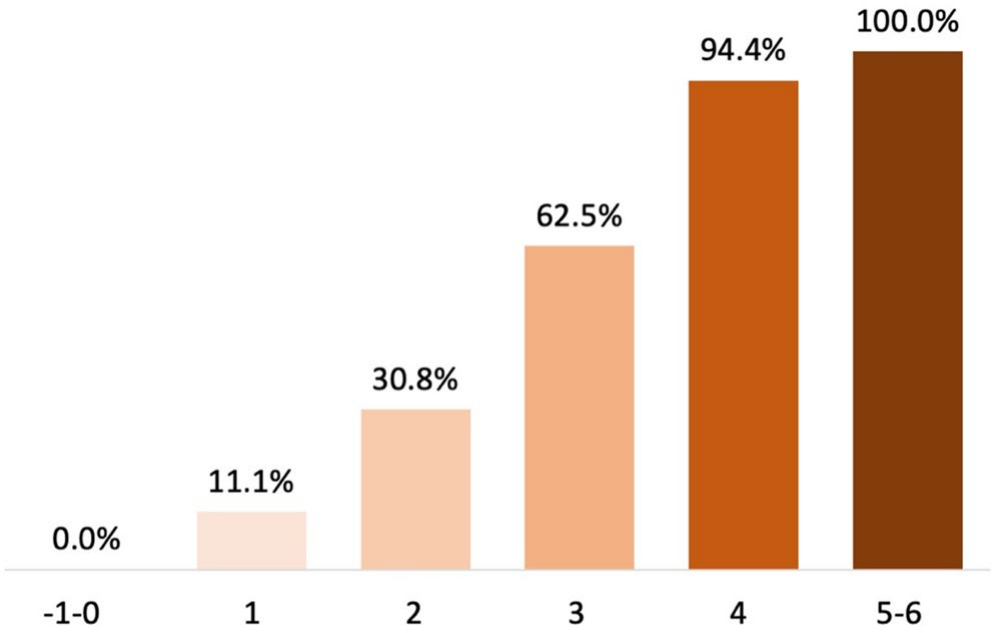
Autoimmune‐associated epilepsy according to the DAME score. Proportions of subjects who developed autoimmune‐associated epilepsy for the different values of the DAME (duration–antibodies–magnetic resonance imaging–electroencephalography) score.

The proportions of subjects who developed autoimmune‐associated epilepsy according to the values of the DAME score are shown in Figure 2. The optimal cutoff value of the score was ≥3 points, yielding a sensitivity of 86.8% (95% CI = 71.9–95.6), a specificity of 87.5% (95% CI = 71.0–96.5), and an overall accuracy of 87.1% (95% CI = 77.0–94.0) for the risk of developing epilepsy. Among subjects with a DAME score of <3 and ≥3 points, five of 33 (15.2%) and 33 of 37 (89.2%; *p* < .001) developed epilepsy, respectively. The positive predictive value was 89.2% (95% CI = 76.6–95.4), and the negative predictive value was 84.9% (95% CI = 71.0–92.8).

## 4. DISCUSSION

In this multicenter, retrospective study of subjects with autoimmune encephalitis presenting as NORSE, more than half of the participants developed epilepsy during the follow‐up. The data support the common notion that NORSE is frequently associated with poor long‐term outcomes, and the evidence suggests that refractory SE is an independent risk factor for unprovoked seizures once the acute phase of the autoimmune encephalitis has resolved. Over a median follow‐up of more than 50 months and with a minimum observation of at least 2 years from the start of immunotherapy, the remaining 45% of the study cohort did not develop epilepsy. These findings highlight the importance of identifying reliable predictors to distinguish patients who will or will not develop autoimmune‐associated epilepsy. In this regard, the model based on the duration of SE, antibody status, localization of MRI abnormalities, and presence of IEDs during follow‐up showed good discriminatory value. The predictive scoring system based on these four factors yielded good sensitivity and specificity, with an overall accuracy of approximately 87%.

Experimental data strongly support the relationship between the duration of epileptic activity and epileptogenesis. In animal models, SE has been shown to result in permanent anatomical changes that induce the reorganization of neural networks.^10^, ^11^, ^12^ In humans, data are more limited and not conclusive. Findings from the current analysis point to the added contribution of SE itself to the process of epileptogenesis and add new insights to prior evidence. In a cohort of 89 nonpediatric patients with SE and without a prior history of epilepsy, 58.7% of survivors showed seizures after a median follow‐up of 10 months.^13^ Epilepsy development was more frequent with longer SE duration, and the association was significant in symptomatic SE due to an acute lesion; most of the patients with cryptogenic or remote/progressive SE developed epilepsy regardless of duration of epileptic activity. SE duration ≥ 24 h was an independent predictor of drug‐resistant epilepsy after convulsive SE.^14^ Duration of SE was longer in patients with NORSE who later developed pharmacoresistant epilepsy (defined as seizures not controlled by two or more ASMs 6 months after discharge) than in those without, and the association remained significant after adjustment for age and sex.^15^ The association between drug‐resistant epilepsy and hippocampal/diffuse cortical atrophy was no longer meaningful after adjustment for SE duration, providing indirect evidence that the duration of seizure activity could be related to brain damage.^15^ In a prospective, single‐center, cohort study enrolling adults with definitive or possible SE, patients with nonreversible peri‐ictal MRI abnormalities had a trend of longer duration of SE compared to those with reversible abnormalities.^16^ Conversely, in a prospective observational cohort study of consecutive adults diagnosed with SE, the rate of seizure recurrence at 3 months after SE onset did not differ between patients who reached the *t*1 and those who reached the longer *t*2 operational dimension of SE; the impact that the short‐term follow‐up may have had on these findings could not be investigated.^17^ In patients with a first SE episode included in a prospective registry, the delay in first‐line treatment emerged as an independent risk factor for the occurrence of remote seizures, whereas SE duration did not; it is noteworthy that an untimely first‐line treatment correlated with SE duration, indicating collinearity between these factors in the population.^18^

The relationship between the presence of antibodies targeting cell surface antigens and a lower risk of autoimmune‐associated epilepsy is consistent with the current framework of the pathophysiology of autoimmune encephalitis. Antibodies against intracellular epitopes cannot reach the intracellular antigens, and cytotoxic T‐cell mechanisms are mainly involved. This process is poorly responsive to treatment and can cause irreversible neuronal damage and increase the risk of associated epilepsy.^19^ Conversely, antibodies directed against cell surface antigens are pathogenic, as they can alter the structure and function of the antigen; this alteration is typically treatment responsive, with a low propensity to result in epileptogenic sequelae.^19^ Within the growing clinical spectrum of autoimmune encephalitis, seronegative forms have become a major subtype.^20^ The incidence and prevalence of seronegative autoimmune encephalitis might be similar to those of seropositive forms.^20^, ^21^ Although autoantibodies not yet identified might account for some cases, seronegative autoimmune encephalitis are thought to be autoimmune disorders with distinct pathomechanisms and clinical features, including a not negligible risk of chronic epilepsy.^19^, ^22^ In a hospital‐based prospective study involving newly diagnosed patients with autoimmune encephalitis, 11.9% of the 67 subjects with 6 months of follow‐up developed epilepsy, and seronegative status was more common in patients who were diagnosed with epilepsy.^23^ In a retrospective observational cohort study including 121 adults with autoimmune encephalitis and a median follow‐up duration of 44 (range = 12–57) months, 29.8% had developed drug‐resistant epilepsy at the last visit.^6^ Drug‐resistant epilepsy was observed in 15.2% of patients with antibody‐positive and 38.7% of patients with antibody‐negative autoimmune encephalitis, and antibody negativity was an independent risk factor for drug‐resistant epilepsy on multivariate regression analysis.

The association between the localization of MRI abnormalities suggestive of autoimmune limbic encephalitis and the risk of chronic epilepsy is consistent with the evidence indicating these areas of the brain are particularly susceptible to epileptogenic processes.^24^ An intimate relationship formed by multiple re‐entrant circuit loops exists between the hippocampus and entorhinal cortex.^24^ These synaptic loops make the temporal lobe vulnerable to the generation of abnormal electrical activity and underlie the predisposition of temporal lobe structures to epileptogenesis.^24^, ^25^, ^26^ Structures including the hippocampus, amygdala, and piriform and entorhinal cortices represent low‐threshold entry points into the limbic seizure circuit.^27^, ^28^ Nodes of this network can represent primary seizure sources and distributors of ictal activity from the driver focus to a broader network.^29^, ^30^ Of note, a prior retrospective cohort study identified T2/FLAIR hyperintensity in the limbic system as a risk factor of drug‐resistant epilepsy in patients with autoimmune encephalitis, and, interestingly, antibody‐negative patients were more likely to have bilateral FLAIR lesions, especially in the limbic system.^6^ IEDs are an electrographic manifestation of excessive hypersynchronization of cortical activity in between seizures and are considered a marker of potentially epileptogenic tissue.^31^ Data provided evidence that interictal discharges represent traveling waves of pathological activity that are similar to their ictal counterparts, and that ictal and interictal discharges arise from common epileptogenic brain regions.^31^ The findings of the current study reinforce the predictive significance of IEDs as an indicator of seizure propensity and expand prior literature suggesting IEDs during follow‐up as a risk factor for the development of epilepsy in patients with autoimmune encephalitis.^32^

This cohort study describes the long‐term seizure outcome in a group of patients with autoimmune encephalitis presenting with NORSE and provides a predictive scoring tool for this specific clinical scenario. The score might be of aid to identify those patients who may require long‐term treatment with ASMs as at high risk to develop autoimmune‐associated epilepsy, thus avoiding the potential consequences of seizure recurrence, and those who may discontinue it after the resolution of the acute phase as at low risk of chronic epilepsy, thus avoiding an unnecessary exposure to medications. The score might also turn out to be useful to individualize the frequency and duration of follow‐up. The main study strengths include the multicenter design and the long‐term follow‐up. Furthermore, the predictive score was based on a few easily available variables. Some limitations need, however, to be acknowledged. Diagnostic workup and treatment regimens followed everyday clinical practice according to the judgment of treating physicians and did not adhere to standardized protocols. Furthermore, the retrospective data collection and the real‐world setting may have introduced potential sources of bias. The proportion of subjects without a diagnosis of epilepsy who were receiving ASMs at the last follow‐up visit was quite modest, yet it cannot be excluded that treatment may have masked the occurrence of seizures, thus underestimating the risk of epilepsy. Although the difference was not statistically significant, there were fewer patients developing autoimmune epilepsy among those who did receive second‐line immune treatments compared to those who did not. The retrospective nature of the study, however, did not allow establishment of any causality, did not allow reaching a conclusion as to whether administration of second‐line immune treatment would be able to reduce the risk of developing post‐NORSE epilepsy, and did not allow excluding that the lack of administration of a second‐line treatment would contribute to the development of autoimmune‐associated epilepsy. The study could not disentangle whether the lower risk of developing epilepsy in patients with autoimmune encephalitis and antibodies against surface antigens was due to a more benign underlying pathophysiology or also influenced by the more common escalation to second‐line immunotherapies. Furthermore, the low number of patients treated with any second‐line regimen prevented the opportunity to explore whether the specific treatments used during the acute phase may have impacted the seizure recurrence. Similarly, the lack of details about the maintenance treatment did not allow exploration of the potential impact of the aggressiveness of immunotherapy after the resolution of SE. The immunotherapy was generally continued after the discharge from the intensive care unit, but the specific regimen, duration, and tapering varied. Treatment options included oral prednisone, periodic intravenous methylprednisolone, intravenous immunoglobulins, rituximab redosing, or steroid‐sparing agents like mycophenolate mofetil. Accordingly, the study did not allow reaching a conclusion as to whether patients treated more aggressively and for a longer time would be less likely to develop epilepsy compared to patients who received immunotherapy for a shorter time frame or only during SE management. Interestingly, according to the study data, there was a substantial delay in the time to immunotherapy with a median increase of 3.5 days or 64% in patients who developed epilepsy compared to those who did not. Although the study could not identify a statistically significant difference, the direction of the findings is consistent with evidence suggesting that the outcome is better the earlier the immunotherapy is started in patients with autoimmune encephalitis.^33^ Despite the rarity of NORSE as a clinical condition, the number of included patients was overall low, limiting the power of statistical analysis. Of note, only a very small minority of participants presented with intracellular antibodies, and most of the cases of seropositive autoimmune encephalitis were associated with antibodies against the N‐methyl‐D‐aspartate receptor, further limiting the generalizability of the findings. Although the discrimination ability of the score was statistically similar in adults and children, larger cohorts could allow exploration of whether potential differences may exist and estimation of the optimal threshold and the yield of sensitivity, specificity, and overall accuracy by age.

## 5. CONCLUSIONS

The role and reciprocal interactions of the immune system and SE‐induced damage in the transition from ictogenesis to epileptogenesis in patients with autoimmune encephalitis are still not completely understood. Through the analysis of long‐term follow‐up data of patients with autoimmune encephalitis presenting with NORSE, the current study provides new clinical insights on the topic and suggests predictors of epilepsy. Additional research is warranted to validate the reliability and predictive accuracy of the proposed scoring system and provide useful complement by adding further variables. It may also be relevant to assess whether the variables included in the DAME score, like the duration of SE, neuroimaging, and EEG findings, can be applied to all NORSE cases, including those that remain cryptogenic. In this regard, a core outcome set for NORSE could facilitate the synthesis of results across studies and improve the quality of the evidence. The continuous exploration and advancements in the characterization of the spectrum of autoimmune encephalitis may improve outcome prediction and offer useful advice to inform clinical practice.

## AUTHOR CONTRIBUTIONS

Simona Lattanzi and Sara Matricardi contributed equally to this paper. Sara Matricardi is responsible for the overall content as the guarantor. Simona Lattanzi, Sara Matricardi, and Flavio Villani conceptualised and designed the study. Simona Lattanzi, Sara Matricardi, Alberto Vogrig, Giada Pauletto, Margherita Nosadini, Stefano Sartori, Federico Massa, Luana Benedetti, Stefano Meletti, Francesca Bisulli, Elena Freri, Francesca Felicia Operto, Silvia Bozzetti, Sara Mariotto, Simone Beretta, Eleonora Rosati, Elisabetta Cesaroni, Carla Marini, Tiziana Granata, and Flavio Villani selected and enrolled patients, critically reviewing all medical charts and records. Sara Matricardi, Simona Lattanzi and Flavio Villani were involved with the dataset and analysis. Simona Lattanzi, Sara Matricardi, and Flavio Villani drafted the manuscript. All collaborators of the Immune Epilepsies Study Group of the Italian League Against Epilepsy (LICE) are responsible for the selection, collection, and diagnostic processes for each case. All authors and collaborators edited the manuscript.

## FUNDING INFORMATION

F.M. was funded by #NEXTGENERATIONEU, funded by the Ministry of University and Research, National Recovery and Resilience Plan, project MNESYS (PE0000006)—"A Multiscale Integrated Approach to the Study of the Nervous System in Health and Disease" (DN. 1553 11.10.2022). F.V. was partially funded by the following research grants: RF‐2021‐12372526 of the Italian Ministry of Health, Ricerca Corrente; and “5 × 1000” of IRCCS San Martino Polyclinic Hospital, Genoa, Italy.

## CONFLICT OF INTEREST STATEMENT

The authors have nothing to disclose related to this study. We confirm that we have read the Journal's position on issues involved in ethical publication and affirm that this report is consistent with those guidelines.

## Supporting information


TABLES S1–S2.

## Data Availability

The data that support the findings of this study are available from the corresponding author upon reasonable request.

## Collaborators

Immune Epilepsies Study Group of the Italian League Against Epilepsy Collaborators: Ruggero Bernabei, Stefania Maria Bova, Silva Cappanera, Luigi Caputi, Edward Cesnik, Francesco Deleo, Lidia Di Vito, Giuseppe D'Orsi, Giovanni Falcicchio, Elisa Fallica, Edoardo Ferlazzo, Lucia Fusco, Matteo Gastaldi, Giada Giovannini, Angela La Neve, Claudio Liguori, Pietro Mattioli, Lorenzo Muccioli, Carlotta Mutti, Angelo Pascarella, Elena Pasini, Daniela Passarelli, Francesca Ragona, Romana Rizzi, Elena Tartara, Valentina Tontini, Alberto Verrotti, Federica Zibordi, Lucia Zinno, Antonio Zito.
