# Supplementary material for: Predicting epilepsy after new onset refractory status epilepticus due to autoimmune encephalitis: The DAME score
Source: Epilepsia. 2025 Dec 30;67(4):1792–801. doi: 10.1002/epi.70081 (PMC13075619; doi:10.1002/epi.70081)
Supplement: Supplementary file 1 — TABLES S1–S2. [file EPI-67-1792-s001.docx]

***Supplementary Appendix***

**p2: Table S1. Autoantibodies identified in the study cohort with proportions of subjects who developed autoimmune-associated epilepsy**

**p3: Table S2. Associations between the items of the DAME score and the development of autoimmune-associated epilepsy according to the antibody-status**

**Table S1. Autoantibodies identified in the study cohort with proportions of subjects who developed autoimmune-associated epilepsy**

| **Antibodies** | **Number of subjects (n=30)** | **Proportion of subjects who developed autoimmune-associated epilepsy** |
| --- | --- | --- |
| Anti - NMDA | 19 (63.3) | 4/19 |
| Anti - LGI1 | 4 (13.3) | 3/4 |
| Anti - MOG | 2 (6.7) | 0/2 |
| Anti - Caspr2 | 1 (3.3) | 0/1 |
| Anti - GABA–A | 1 (3.3) | 1/1 |
| Anti - GAD | 1 (3.3) | 1/1 |
| Anti - Hu | 1 (3.3) | 0/1 |
| Anti - Titin | 1 (3.3) | 0/1 |

Abbreviations: Caspr2=contactin-associated protein-like 2, GABA-A=γ-aminobutyric acid type A, GAD=glutamic acid decarboxylase, LGI1=leucine-rich glioma inactivated, MOG= myelin oligodendrocyte glycoprotein, NMDA=N-methyl-D-aspartate.

**Table S2. Associations between the items of the DAME score and the development of autoimmune-associated epilepsy according to the antibody-status**

| **Antibody positive (n=30)** | | | |
| --- | --- | --- | --- |
|  | **Autoimmune-associated epilepsy** | | **OR (95% CI); p value** |
|  | **No (n=21)** | **Yes (n=9)** |  |
| Duration of status epilepticus ≥10 days | 11 (52.4) | 9 (100.0) | ^a^Not estimable |
| **^*^**MRI bitemporal abnormalities | 1 (4.8) | 4 (44.4) | 16.00 (1.45-176.45); p=0.024 |
| EEG interictal epileptiform discharges | 4 (19.1) | 6 (66.7) | 8.50 (1.46-49.54); p=0.017 |
| **Antibody negative (n=40)** | | | |
|  | **Autoimmune-associated epilepsy** | | **OR (95% CI); p value** |
|  | **No (n=11)** | **Yes (n=29)** |  |
| Duration of status epilepticus ≥10 days | 6 (54.6) | 27 (93.1) | 11.25 (1.75-72.50); p=0.011 |
| **^*^**MRI bitemporal abnormalities | 0 (0.0) | 11 (37.9) | ^a^Not estimable |
| EEG interictal epileptiform discharges | 3 (27.3) | 25 (86.2) | 16.67 (3.06-90.82); p=0.001 |

**^*^**T2/FLAIR hyperintensity compatible with inflammation. ^a^Outcome perfectly predicted.

Data are n (%) unless otherwise specified.

Abbreviations: CI=confidence interval, FLAIR=fluid-attenuated inversion recovery, OR=odds ratio.
